# Understanding maternal mortality in women with obesity and the role of care they receive: a national case-control study

**DOI:** 10.1038/s41366-020-00691-4

**Published:** 2020-10-22

**Authors:** Monica Saucedo, Ana Paula Esteves-Pereira, Lucile Pencolé, Agnès Rigouzzo, Alain Proust, Marie-Hélène Bouvier-Colle, CNEMM study group, Dominique Chassard, Henri Cohen, Michel Dreyfus, Jean-Claude Ducloy, Irina Guseva-Canu, Jean-Pierre Laplace, Véronique Le Guern, Sylvie Leroux, Estelle Morau, Claire Rondet, Mathias Rossignol, Véronique Tessier, Éric Verspyck, Philippe Weber, Laurent Zieleskiewicz, Catherine Deneux-Tharaux

**Affiliations:** 1 Université de Paris, CRESS, Obstetrical, Perinatal and Pediatric Epidemiology Research Team, EPOPé, INSERM, INRA, DHU Risks in pregnancy, Paris, France; 2 Department of Epidemiology and Quantitative Methods in Health, Sérgio Arouca National School of Public Health, Oswaldo Cruz Foundation, Rio de Janeiro, Brazil; 3 Department of Obstetrics and Gynecology, Armand Trousseau Hospital, Assistance publique des hôpitaux de Paris, Paris, France; 4 Department of Anesthesiology, Armand Trousseau University Hospital, Assistance publique des hôpitaux de Paris, Paris, France; 5 Department of Obstetrics and Gynecology, Hôpital Privé d’Antony, Antony, France; 6 Department of Anesthesia and Intensive Care, Femme Mère Enfant Hospital, Hospices Civiles de Lyon, Lyon, France; 7 Department of Obstetrics and Gynecology, Institut Mutuliste Montsouris, Paris, France; 8 Department of Obstetrics and Gynecology, CHU de Caen, Caen, France; 9 Department of Anesthesia, Hôpital privé Villeneuve d’Ascq, Villeneuve d’Ascq, France; 10 Non-communicable diseases and trauma division, Santé publique France, Saint-Maurice, France; 11 Department of Obstetrics and Gynecology, Polyclinique Bordeaux Nord Aquitaine, Bordeaux, France; 12 Centre de Référence Maladies Auto-Immunes et Systémiques Rares, Service de Médecine Interne Pôle Médecine, Hôpital Cochin, AP-HP, Paris, France; 13 Department of Obstetrics and Gynecology, Centre Hospitalier Annecy-Genevois, Annecy, France; 14 Department of Anaesthesia, CHU de Nimes, Nimes, France; 15 Centre for Epidemiology on Medical Causes of Death (CépiDc-Inserm), Le Kremlin-Bicêtre, France; 16 Department of Intensive Care, Hôpital Lariboisière, Assistance Publique Hôpitaux de Paris, University of Paris, Paris, France; 17 DHU Risques et Grossesse, Maternité Port Royal, Hôpitaux Universitaires Paris Centre, Hôpital Cochin, AP-HP, Paris, France; 18 Department of Obstetrics and Gynecology, CHU de Rouen, Rouen, France; 19 Department of Obstetrics and Gynecology, Centre Hospitalier de Mulhouse, Mulhouse, France; 20 Département Anesthésie-Réanimation, Assistance publique-Hôpitaux de Marseille, Hôpital Nord, Marseille, France

**Keywords:** Epidemiology, Obesity, Risk factors

## Abstract

**Objective:**

Obesity has significant implications for the health of pregnant women. However, few studies have quantified its association with maternal mortality or examined the relevant underlying causes and the role of care, although this remains the most severe maternal outcome. Our objectives were to quantify the risk of maternal death by prepregnancy body mass index and to determine whether obesity affected the quality of care of the women who died.

**Desing:**

This is a national population-based case–control study in France. Cases were 364 maternal deaths from the 2007–2012 National Confidential Enquiry. Controls were 14,681 parturients from the nationally representative 2010 perinatal survey. We studied the association between categories of prepregnancy BMI and maternal death by multivariable logistic regression, estimating adjusted odds ratios and 95% confidence intervals, overall and by specific causes of death. Individual case reviews assessed the quality of care provided to the women who died, by obesity status.

**Results:**

Compared with women with normal BMI, underweight women (<18.5 kg/m^2^) had an adjusted OR of death of 0.75 (95% CI, 0.42–1.33), overweight women (25–29.9 kg/m^2^) 1.65 (95% CI, 1.24–2.19), women with class 1 obesity (30–34.9 kg/m^2^) 2.22 (95% CI, 1.55–3.19) and those with class 2–3 obesity (≥35 kg/m^2^) 3.40 (95% CI, 2.17–5.33). Analysis by cause showed significant excess risk of maternal death due to cardiovascular diseases, venous thromboembolism, hypertensive complications and stroke in women with obesity. Suboptimal care was as frequent among women with (35/62, 57%) as without obesity (136/244, 56%), but this inadequate management was directly related to obesity among 14/35 (40%) obese women with suboptimal care. Several opportunities for improvement were identified.

**Conclusions:**

The risk of maternal death increases with BMI; it multiplied by 1.6 in overweight women and more than tripled in pregnant women with severe obesity. Training clinicians in the specificities of care for pregnant women with obesity could improve their outcomes.

## Introduction

As obesity (body mass index, that is, BMI, ≥30 kg/m^2^) increases in both low- and high-income countries [1], so does its prevalence in pregnant women. It affects about 20% of those in England [2] and 25% in the United States [3, 4]. In France, the proportion of parturient women with prepregnancy obesity grew from 7.5% in 2003 to 11.8% in 2016 [5]. These trends have aggravated concerns about its associated adverse maternal and neonatal outcomes.

Maternal obesity is a risk factor for pregnancy-related disorders, including gestational hypertension, preeclampsia, and gestational diabetes [6–8]. The risk of maternal complications is also increased during the postpartum period, including postpartum haemorrhage [8, 9], postpartum infection, and longer post-delivery hospital stays [10]. Recent studies show that obesity is associated with severe maternal morbidity [8, 11, 12], but fewer investigations have explored or quantified its association with maternal mortality or examined the relevant underlying causes of death, although this remains the most severe maternal outcome [13–15]. Documentation of the role in this association of these women’s other characteristics and of the quality of care they receive remains sparse. To prevent these severe outcomes, we must examine, beyond mortality, whether obesity results in gaps of treatment of pregnancy complications and if specific aspects of medical care for these women require improvement.

Our first aim was to quantify the risk of maternal death independently associated with prepregnancy obesity, overall and by underlying cause of death, after controlling for individual confounders. Second, and the novelty of our study, we sought to determine whether their obesity affected the quality of care of the women who died and identify opportunities to improve care.

## Materials and methods

This population-based case–control study used cases and controls from nationwide surveys in France.

### Cases

Cases came from the French National Confidential Enquiry into Maternal Deaths (ENCMM, *Enquête Confidentielle sur les Morts Maternelles*) [16, 17]. This permanent survey has studied all deaths since 1996 of women during pregnancy or within 1 year of its end. The sources used to identify these deaths and the procedures for investigating and assessing them have been described in detail [16, 18]. In brief, besides spontaneous notifications by clinicians, deaths are identified from three sources: (1) death certificates of women of reproductive age; (2) computer-based national linkage of the death register with the birth register, from death and birth certificates; and (3) hospital discharge databases. Two assessors (an obstetrician or midwife and an anaesthetist) conduct a confidential enquiry of each pregnancy-associated death identified, collecting relevant clinical information about the woman and her death through interviews and review of the medical records and autopsy reports. The ENCMM national expert committee analyses the anonymised deaths and consensually determines for each: (1) its underlying cause, i.e., the illness or complication triggering the chain of events until death; (2) whether it was a maternal death, defined as a woman’s death during pregnancy or within 1 year of its end, regardless of its duration and site, from any cause related to or aggravated by the pregnancy or its management (but not accidental or incidental); and (3) the quality of care provided (optimal or non-optimal) and the preventability of maternal deaths. This study includes maternal deaths identified by the ENCMM for 2007–2012 (the six most recent years available) in mainland France (*n* = 441). After the exclusion of 77 maternal deaths before 22 weeks’ gestation (during pregnancy or post-abortion), for consistency with the definition of controls below, this analysis included 364 maternal deaths.

### Controls

Controls came from a representative sample of births in France, the 2010 French National Perinatal Survey (NPS), a repeated cross-sectional study conducted periodically since 1995 to produce statistics about perinatal indicators [19]. These surveys cover all live- and stillbirths at or after 22 weeks of gestation or with a birth weight of at least 500 g in every maternity unit in France for a complete 1-week period (1/52nd of births in France). Midwives interview mothers before discharge to obtain data on maternal social and demographic characteristics and prenatal care. Data about the pregnancy, delivery and newborns are collected from medical records. The 2010 version of the NPS, in the middle of the 2007–2012 case inclusion period, served as the source population for the control women in this analysis (*n* = 14 681). Its methods have been detailed elsewhere [19]. By definition, the control group should not include women who died in the postpartum period. Although it is possible that some died after discharge, the reported maternal mortality ratio in France is low enough (10.3/100,000 live births 2007–2012) to consider the number of such cases, about one or two expected maternal deaths after discharge among the 14,681 women, negligible [17].

Ethical approval for both the ENCMM and NPS surveys was granted by the French Commission on Information Technology and Liberties.

The primary predictor variable was categorical prepregnancy body mass index (BMI; kg/m2) as underweight: BMI < 18.5; normal weight: BMI 18.5–24.9; overweight: BMI 25–29.9; class 1 obesity: BMI 30–34.9; class 2–3 obesity: BMI ≥ 35. BMI was calculated from self-reported prepregnancy weight and height in the medical records of cases and controls. BMI categories for class 2 and class 3 obesity were combined because of the small number of cases in these groups. The precise BMI for some women was not available because either weight or height or both were missing, but the prepregnancy obesity status (yes/no) was reported from another questionnaire item. The following covariates were examined: mother’s country of birth (France or other European country, North Africa, sub-Saharan Africa, other), mother’s age (≤24, 25–29, 30–34, 35–39, ≥40 years), educational level of zip-code of residence (proportion of adults 20–45 years who did not complete high school, grouped into quintiles based on data available from the French census) as a proxy for socioeconomic status, severe pre-existing chronic conditions (a composite binary variable including hypertension, diabetes, cardiac diseases, and other notable chronic conditions), previous deliveries (nulliparous, only vaginal deliveries, one or more caesarean deliveries), multiple pregnancy and median gestational age (weeks).

### Statistical analysis

The characteristics of cases and controls were compared by chi-square or Wilcoxon tests. The association between prepregnancy BMI and maternal mortality was assessed by univariate and multivariable logistic regression models. We selected potential confounders from the literature and the univariate analysis and adjusted for them. Pre-existing chronic conditions were not included in the main adjusted models because they were considered potential mediators rather than confounders of the link between obesity and maternal death [20]. The analysis by causal condition used the main cause of death, exclusive categorization, determined by the ENCMM expert committee. To assess the cause-specific risk of maternal mortality associated with maternal body mass, we used a binary obesity (yes/no) exposure variable, because of the limited numbers of maternal deaths by cause. Crude and adjusted odds ratios were calculated with 95% confidence intervals. Interactions between obesity and covariables were systematically tested.

The proportion of women with missing information was 15.9% for obesity status and 33.0% for BMI among maternal deaths, and 7.0 and 7.5% respectively among controls. We handled missing values by multiple imputation with chained equations (10 datasets) on the missing-at-random hypothesis [21]. The multiple imputation prediction model was composed of the outcome variable, prepregnancy BMI, maternal age, country of birth, quintile of zip-code education level, pre-existing chronic conditions, previous deliveries, multiple pregnancy and duration of pregnancy. Results are presented with imputed data.

Post-hoc calculations showed that with a significance level of 5% and obesity prevalence of 10% among controls, this sample would have >90% power to detect a doubled risk of death in obese versus non-obese women (OR ≥ 2.0).

We performed several sensitivity analyses. The first tested the impact of adjusting for severe chronic conditions before pregnancy together with confounders in the multivariate logistic regression model; although we primarily considered this variable an intermediate factor (see above), it might also affect maternal risk directly, regardless of obesity status. Second, we assessed the relevance of the multiple imputation for missing data and performed an analysis of the association between BMI and maternal mortality with non-imputed data.

The second part of the analysis was restricted to women who died. Here we compared the proportion of suboptimal care, assessed by the ENCMM expert committee, by obesity status. To isolate the components of suboptimal care directly related to obesity, we reviewed each death of an obese woman with suboptimal care, relying on the ENCMM committee determinations given the absence of specific clinical guidelines in France for managing pregnancy in women with obesity.

Analyses used STATA 14.0 (StataCorp LP, College Station, TX, USA).

## Results

Compared with controls, cases were more often born outside France (or Europe), older, and more likely to have severe chronic conditions before pregnancy, at least one previous caesarean delivery and a multiple pregnancy (Table 1). The distribution of BMI differed significantly between cases and controls. The proportion of overweight and obesity was significantly higher among women who died than among controls (Table 2).

**Table 1:** Characteristics of women who died and controls.

	Women who died		Controls		*P* value^a^
	*n*	%	*n*	%	
	364	100.0	14,681	100.0	
Country of birth (*n* = 354 and 14,135)					
France or other European country	267	75.4	12,094	85.6	<0.001
North Africa	32	9.0	1002	7.1	
Sub-Saharan Africa	35	9.9	608	4.3	
Other	20	5.7	431	3.1	
Age (years) (*n* = 364 and 14,530)					
≤24	42	11.5	2474	17.0	<0.001
25–29	81	22.3	4817	33.2	
30–34	102	28.0	4452	30.6	
35–39	99	27.2	2279	15.7	
≥40	40	11.0	508	3.5	
Educational level of zip-code of residence (proportion of adults 20–45 years who did not complete high school) (*n* = 358 and 14,094)					
1st quintile (<36%)	55	15.4	2824	20.0	0.08
2nd quintile (35%–45%)	80	22.3	2835	20.1	
3rd quintile (45%–51%)	64	17.9	2853	20.2	
4th quintile (51%–57%)	75	20.9	2805	19.9	
5th quintile (>57%)	84	23.5	2777	19.7	
Severe chronic conditions before pregnancy					
Any (*n* = 313 and 14,318)	96	30.7	529	3.7	<0.001
Hypertension (*n* = 305 and 14,484)	22	7.23	150	1.0	<0.001
Diabetes (*n* = 305 and 14,491)	23	7.5	71	0.5	<0.001
Cardiopathies (*n* = 306 and 14,465)	21	6.9	51	0.4	<0.001
Another notable pre-existing chronic condition (*n* = 304 and 14,545)	45	14.8	276	1.9	<0.001
Previous deliveries (*n* = 311 and 14,410)					
No	105	33.8	6302	43.7	<0.001
Yes, only vaginal	117	37.6	6524	45.3	
Yes, one or more Caesareans	89	28.6	1584	11.0	
Multiple pregnancy (*n* = 356 and 14,654)	17	4.8	224	1.5	<0.001
Median gestational age (weeks [IQR])	38.0	[33.0–39.0]	39.0	[38.0–40.0]	<0.001

*IQR* interquartile range.

^a^Chi-square or Wilcoxon test.

**Table 2:** Risk of maternal mortality according to prepregnancy body mass index.

Prepregnancy body mass index (kg/m2)	Women who died (*n* = 364)%	Controls (*n* = 14,681)%	Crude OR (95% CI)	Adjusted OR^a^ (95% CI)
Underweight (<18.5)	4.01	8.3	0.65 (0.37–1.15)	0.75 (0.42–1.33)
Normal (18.5–24.9)	48.2	64.2	1	1
Overweight (25–29.9)	25.0	17.7	1.88 (1.43–2.47)	1.65 (1.24–2.19)
Class 1 obesity (30–34.9)	13.2	6.8	2.61 (1.83–3.72)	2.22 (1.55–3.19)
Class 2–3 obesity (≥35)	9.5	3.0	4.18 (2.83–6.83)	3.40 (2.17–5.33)

*OR* odds ratio, 95% *CI* 95% confidence interval.

^a^Logistic regression models adjusted for country of birth, maternal age, zip-code education level quintile, parity including previous caesarean delivery; with multiple imputation for missing data.

After adjustment for confounders, the risk of maternal death increased with categories of BMI, with an adjusted OR of 1.65 for overweight women and of 3.40 for women with class 2–3 obesity, compared with women with a normal BMI. The risk in underweight women did not significantly differ from that of normal-weight women. The analysis with non-imputed data provided similar results (Table S1). The addition of pre-existing medical conditions as covariates in the multivariable model provided similar results although the associations were attenuated (Table S2).

We further analysed the risk of cause-specific maternal mortality associated with prepregnancy obesity. Women with obesity had a statistically significantly higher risk of maternal death from cardiovascular disease (CVD), thromboembolism, hypertensive complications and stroke, after adjustment for confounders, compared with non-obese women (Table 3).

**Table 3:** Risk of cause-specific maternal mortality associated with prepregnancy obesity.

Cause of death^a^	Women who died (*n* = 364)%		Controls (*n* = 14,681)%		Crude OR (95% CI)	Adjusted OR^b^ (95% CI)
	Obese	Non-obese	Obese	Non-obese		
All-cause mortality	22.7	77.3	9.8	90.2	2.72 (2.10–3.54)	2.37 (1.81–3.10)
Cardiovascular	33.1	66.9			4.52 (2.35–8.73)	4.55 (2.33–8.87)
Thromboembolism	26.6	73.4			3.30 (1.35–8.06)	2.87 (1.18–6.97)
Hypertensive complication	24.8	75.2			3.04 (1.23–7.50)	2.86 (1.14–7.15)
Stroke	26.2	73.8			3.04 (1.14–8.11)	2.84 (1.06–7.69)
Amniotic fluid embolism	21.7	78.3			2.57 (1.19–5.52)	2.10 (0.97–4.57)
Haemorrhage	20.5	79.5			2.37 (1.12–5.02)	1.79 (0.83–3.86)
Infection	15.8	84.2			1.68 (0.48–5.96)	1.46 (0.40–5.28)

*OR* odds ratio, *95%* *CI* 95% confidence interval.

^a^Assessed by the national expert committee, exclusive categorization.

^b^Logistic regression models adjusted for country of birth, maternal age, parity and previous caesarean delivery; with multiple imputation for missing data.

The ENCMM expert committee considered it had sufficient information to judge the quality of care for 306 of the 364 maternal deaths. The proportion of suboptimal care did not differ between the obese and non-obese women who died (35/62, 57%, vs 136/244, 56%, *P* = 0.9). Among the 35 women with obesity who died after receiving suboptimal care, this care involved factors related to obesity for 14/35 (40%), 8 with class 1 obesity and 6 with class 2–3. Thes causes of these 14 deaths were 4 pulmonary embolisms 2 uterine ruptures, 2 amniotic fluid embolisms, 2 cardiomyopathies, 2 brain anoxia secondary to complicated endotracheal intubation, 1 sepsis and 1 HELLP syndrome. Specifically, there were technical difficulties with clinical examinations or procedures for diagnosis or treatment (abdominal ultrasound, venous access, intubation, tracheotomy, extracorporeal membrane oxygenation) and treatment underdosing (antibiotics, anticoagulants). Table 4 reports these factors. The two vignettes in supplementary material briefly illustrate some of these factors

**Table 4:** Specific factors of suboptimal care involving obesity among obese women who died with suboptimal care (*n* = 14).

Type of obesity-related suboptimal care^a^	Description	Number of women concerned
A. Failures in preconceptional evaluation	Inadequate assessment of concomitant risk of obesity with severe comorbidities	1
B. Inadequate antenatal care	Underestimation of clinical signs (tiredness, dyspnoea, pain) systematically and wrongly attributed to obesity and leading to misdiagnosis of the condition that led to death	6
C. Failures in diagnostic or therapeutic procedures	Technical difficulties in obese patients: medical imaging procedures, venous access, endotracheal intubation, extracorporeal membrane oxygenation	8
D. Underdosed treatment	Insufficient dose of antibiotics or anticoagulants according to body mass index	5

^a^Non-exclusive categories.

## Discussion

### Principal findings of the study

This national study shows that after adjustment for confounders the risk of maternal death increases as BMI rises, multiplying by 1.6 in women with overweight and more than tripling in women with severe obesity, compared with normal weight women. This excess risk is mainly explained by increased maternal mortality due to CVDs, hypertensive complications, thromboembolism and stroke. Suboptimal care among the women with obesity who died often involved inadequate management directly related to obesity; opportunities for improvement therefore exist.

### Interpretation

Although several studies have addressed the association between obesity and diverse adverse pregnancy outcomes, evidence for the link with maternal mortality has been sparse and inconsistent. A California study found prepregnancy obesity more frequent among women who died than among a reference parturient population (30 vs 16%); [14] in Florida, relative ratios of maternal death showed a rising trend with severity of obesity (from a 2.8-fold increase for class 1 to sevenfold for class 3, compared with women with normal weight) [22]. Confounders may, however, have biased these crude associations because neither study considered any of the women’s other individual characteristics. In contrast, a UK national case–control study assessing risk factors for maternal mortality found that it was not significantly associated with obesity after controlling for other individual characteristics, including pre-existing chronic conditions [23]. Because some comorbidities are likely consequences of obesity and thus intermediate rather than confounding factors in this association, their analysis strategy might have masked obesity’s actual role. Our analysis treated pre-existing chronic conditions as an intermediate factor instead of a confounder, and our sensitivity analysis, which included severe chronic conditions before pregnancy in the multivariable model, showed an attenuated but still significant association between BMI and maternal mortality.

The risk of maternal mortality from CVD was four times higher in women with than without obesity. Although the link between obesity and CVD in the general adult population is well known [24–26], an associated increase in mortality risk is less clear [27, 28]. This issue has been studied still less among pregnant women. A population-based study in the state of Washington found a risk of severe cardiac morbidity three times higher among women with class 3 obesity than among those without obesity during hospitalisation for delivery [11]. While this association might indirectly reflect the higher prevalence of chronic conditions in women with obesity, the excess risk for cardiovascular mortality remained significant in our sensitivity analysis after controlling for severe chronic conditions before pregnancy. This adverse outcome may reflect a reduced capacity to respond to pregnancy-induced cardiovascular changes, including cardiovascular compliance and maximum heart rate, among other biological effects, induced by obesity [29, 30]. In our study, one-third of the women with obesity who died of CVD had been diagnosed with it before pregnancy. Risk stratification is needed for these women before or early in pregnancy, together with joint management by a cardiologist and an obstetrician. Inversely, CVD developed or was revealed during pregnancy or the postpartum for the remaining two-thirds. This suggests that clinicians involved in antenatal care of women with obesity must pay particular attention to screening for cardiovascular clinical signs during pregnancy and after delivery.

Women with obesity also had a risk almost three times higher than those without it of maternal death from thromboembolism, hypertensive complications and stroke. The incidence of these conditions is known to be increased in women with obesity [6, 31–33]. Our analysis shows that this susceptibility persists along the continuum of morbidity and translates into increased mortality. In addition, factors related to the quality of care provided to women with obesity might aggravate this disparity; these include the adequacy of blood pressure monitoring among women with obesity or the failure to take body mass into account when adjusting the dose of thromboprophylaxis.

One original aspect of our study is the case review conducted to assess care received by women with obesity and identify opportunities for improvement. This approach can and should be developed beyond assessing these women’s risk of severe maternal complications. This detailed case review suggests that some of these outcomes might have been prevented by improvement of some aspects of the care they received during pregnancy and delivery. Our results, by showing that improvement opportunities in obese women who died often involve aspects of care directly related to obesity, highlight the need to customise their care before and during pregnancy and in the peripartum period. This has several implications for care improvement: first, screening for clinical signs suggestive of cardiac conditions such as dyspnoea should be part of routine prenatal care in pregnant women with obesity; second, initial and continuing training of professionals involved in prenatal care should ensure that they know the specificities of clinical management of pregnant women with obesity and help them to anticipate challenging medical procedures (diagnostic, anaesthesiological, or surgical) and dosing strategies for specific treatments, such as anticoagulants or antibiotics. Those results provide scientific support to some of the recommendations for care of women with obesity (such as the recent “Care of Women with Obesity in Pregnancy” NICE guideline), and also highlight some aspects not mentioned in those recommendations and that may usefully be added, in particular the need for careful screening for cardiac clinical signs during pregnancy and after delivery [34].

### Strengths

Several strengths characterised our study. We assessed the risk of maternal mortality according to BMI class with cases and controls from nationwide population-based sources providing comprehensive individual data for pregnant women. The availability of detailed data on maternal deaths enabled further analysis of the care these women received so that we could isolate the aspects of inadequate management directly related to obesity and propose areas for improvement.

### Limitations

This study has some limitations. First, some maternal BMI data were missing. Nonetheless, we were able to include all women in the analysis by using multiple imputation for missing data, and the results of the sensitivity analysis with non-imputed data were similar to those with imputed data. Another limitation, due to case number constraints, was the need to study obesity with a binary variable, rather than classes of BMI, in the risk assessment by specific causes of maternal mortality. Because this binary obesity variable combines women with underweight, normal weight, and overweight women in the same reference class, it may result in underestimating the association studied, but does not challenge our main conclusions. Additionally, prepregnancy BMI may be inaccurate because height and weight were self-reported. Nevertheless, a recent meta-analysis showed that self-reported weight of women of reproductive age differs only slightly from direct measures [35]. Finally, beyond BMI, gestational weight gain, a recently highlighted risk factor for adverse pregnancy outcomes [36], was not taken into account in our analysis, because related data were not available. Nonetheless, a noteworthy study from the state of Washington did not show any substantial modification in the associations between BMI and maternal outcomes after controlling for gestational weight gain [11].

## Conclusion

The risk of maternal death increases with BMI. Public health interventions promoting lifestyle changes for obesity management remain a major challenge. Identifying modifiable factors related to the health care provide to women with obesity might produce a more positive effect. The opportunities for improvement identified in this study in a substantial proportion of women emphasise the need for optimal screening and management of CVD in women with obesity before and during pregnancy and for training clinicians in the specificities of their care.

## Supplementary information

Suplementary tables and vignettes
