# Supplementary material for: Understanding maternal mortality in women with obesity and the role of care they receive: a national case-control study
Source: Int J Obes (Lond). 2020 Oct 22;45(1):258–65. doi: 10.1038/s41366-020-00691-4 (PMC7752756; doi:10.1038/s41366-020-00691-4)
Supplement: Supplementary file 1 — Suplementary tables and vignettes [file 41366_2020_691_MOESM1_ESM.docx]

**Supplementary Tables**

| **Table S1 - Risk of maternal mortality according to prepregnancy body mass index. Multivariable analysis with non-imputed data** | | | | | |
| --- | --- | --- | --- | --- | --- |
| **Prepregnancy body mass index (kg/m2)** | Women who died (244)  % | Controls (13 581)  % | Crude OR  (95% CI) |  | Adjusted OR^a^  (95% CI) |
| Underweight (<18.5) | 4.1 | 8.2 | 0.65 (0.34-1.26) |  | 0.62 (0.30-1.29) |
| Normal (18.5- 24.9) | 49.6 | 64.9 | 1 |  | 1 |
| Overweight (25-29.9) | 22.1 | 17.0 | 1.70 (1.23-2.35) |  | 1.53 (1.10-2.13) |
| Class 1 obesity (30-34.9) | 14.3 | 6.8 | 2.71 (1.84-4.04) |  | 2.27 (1.51-3.40) |
| Class 2-3 obesity (≥35) | 9.8 | 3.1 | 4.17 (2.66-6.54) |  | 2.95 (1.80-4.90) |
| OR, odds ratio; 95% CI, 95% confidence interval | | | | | |
| ^a^ Logistic regression models adjusted for country of birth, maternal age, zip-code education level quintile, parity including previous caesarean delivery; Controls n=13 053, Cases n=231 | | | | | |

| **Table S2 - Risk of maternal mortality according to prepregnancy body mass index. Multivariable analysis adjusting for pre-existing medical conditions.** | | | | | |
| --- | --- | --- | --- | --- | --- |
| **Prepregnancy body mass index (kg/m2)** | Women who died (364)  % | Controls (14 681)  % | Crude OR  (95% CI) |  | Adjusted OR^a^  (95% CI) |
| Underweight (<18.5) | 4.1 | 8.3 | 0.65 (0.37-1.15) |  | 0.79 (0.44-1.41) |
| Normal (18.5- 24.9) | 48.2 | 64.2 | 1 |  | 1 |
| Overweight (25-29.9) | 25.0 | 17.7 | 1.88 (1.43-2.47) |  | 1.53 (1.14-2.05) |
| Class 1 obesity (30-34.9) | 13.2 | 6.7 | 2.61 (1.83-3.72) |  | 1.80 (1.23-2.62) |
| Class 2-3 obesity (≥35) | 9.5 | 3.0 | 4.18 (2.83-6.83) |  | 2.17 (1.35-3.50) |
| OR, odds ratio; 95% CI, 95% confidence interval | | | | | |
| ^a^ Logistic regression models adjusted for country of birth, maternal age, zip-code education level quintile, parity including previous caesarean delivery, and pre-existing medical conditions; with multiple imputation for missing data. | | | | | |

**Examples of suboptimal care involving obesity among obese women who died**

**The two vignettes below briefly illustrate some of the factors described in table 4:**

*A case of inadequate risk assessment and underdosed treatment*: A 35 year old woman with a BMI of 44kg/m2 and a previous caesarean section delivered by uneventful repeated caesarean section at 40 weeks of gestation. She received 40mg of enoxaparin per day only during hospital stay (five days), and was home discharged without anticoagulant treatment. She collapsed 20 days after delivery and died one hour after despite cardiopulmonary resuscitation. On autopsy, massive bilateral pulmonary embolism was found.

*A case of technical difficulties leading to misdiagnosis ad delayed tetament*: A 35 year old woman with a BMI of 46 kg/m2 with two previous caesarean sections collapsed at home at 35 weeks of gestation. She was quickly transferred o emergency ward; at admission her blood pressure was 120/70 mmHg and Glasgow Coma Score = 12. The physical and ultrasound examination of her uterus was difficult due to the thick abdominal wall and the initial assessment concluded a foetal death with no other abnormal findings. One hour later a repeated ultrasound exam found an abruptio placentae. At emergency laparotomy, a uterine rupture with massive hemoperitoneum was found. During surgery, a first and second cardiac arrests occurred. With difficulties, a femoral central venous access was installed, which delayed massive blood transfusion. Cardiac activity was not restored and death was pronounced 5 hours after admission.
